# Investigations of Enteric-Coated Tablet Propyl Gallate-Induced Nephrotoxicity in Beagles as well as Human and Dog Renal Proximal Tubule Epithelial Cells

**DOI:** 10.1021/acsptsci.4c00563

**Published:** 2025-04-04

**Authors:** Si Mou, B. Timothy Hummer, Jiaqi Yuan, Yue Huang, Meina Liang, Raffaella Faggioni, Lorin K. Roskos, Anton I. Rosenbaum

**Affiliations:** † Integrated Bioanalysis, Clinical Pharmacology & Safety Sciences, 468087 R&D, AstraZeneca, South San Francisco, California 94080, United States; ‡ Cardiovascular, Renal and Metabolism Safety, Clinical Pharmacology & Safety Sciences, R&D, AstraZeneca, Gaithersburg, Maryland 20878, United States; § Clinical Pharmacology & Safety Sciences, R&D, AstraZeneca, South San Francisco, California 94080, United States

**Keywords:** propyl gallate, enteric-coating, nephrotoxicity, glutathione, dog, MEDI7219

## Abstract

During nonclinical development of an oral formulation for a glucagon-like peptide-1 (GLP-1) receptor agonist, MEDI7219, toxicology studies revealed that propyl gallate (PG), when administered in enteric-coated (EC) tablets, led to nephrotoxicity in beagles. While PG has been widely used in food and cosmetics as an antioxidant, understanding of its toxicology, metabolism, and pharmacokinetics has been rarely discussed. To elucidate the nephrotoxicity observed after administration of PG in an EC tablet formulation, we employed dog and human renal proximal tubule epithelial cells (RPTECs). We observed greater cytotoxicity to PG in dog RPTECs compared to human cells and greater increases in response to PG treatment of glutathione in human cells compared to dog cells. Glutathione elevation is a common response to detoxify xenobiotics, especially ones that produce free radicals such as PG. We hypothesize that glutathione in human RPTECs was elevated to detoxify PG, but not in dog RPTECs, leading to greater cytotoxicity for dog RPTECs. However, a subsequent study in dogs demonstrated that the oral administration of PG in a non-EC capsule did not result in renal toxicity, suggesting the physiological response to PG is modulated by the mode of absorption and a blunted glutathione response may not completely explain the PG-related renal toxicity observed in dogs. Furthermore, to characterize the pharmacokinetics and metabolism of PG we developed a 10-plex, highly sensitive and robust LC-MS/MS-based quantification method for PG and its phase-I and phase-II metabolites. The methods were employed to support preclinical dog studies and clinical study (NCT03362593).

## Graphical abstract

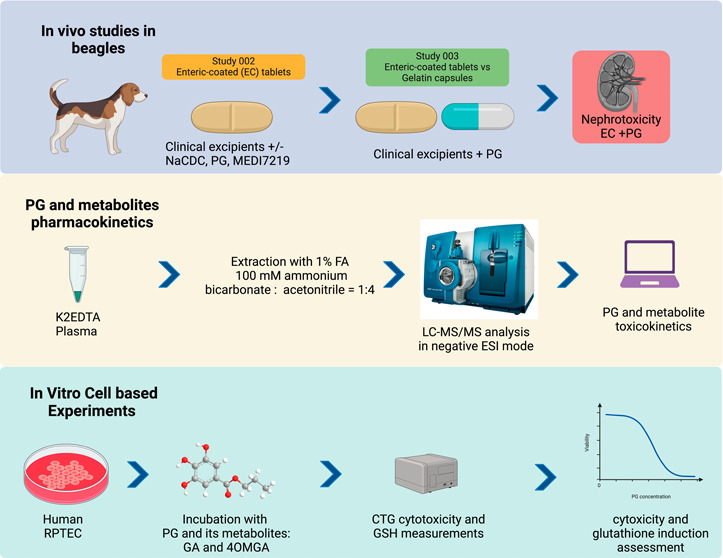

MEDI7219 is a bis-lipidated glucagon-like peptide-1 (GLP-1) analog with enhanced systemic circulation time due to increased reversible binding to albumin. It was being developed as an oral formulation to improve glycemic control and body weight loss in patients with Type 2 diabetes.^1,2^ Currently, most GLP-1 receptor agonists are administered via subcutaneous (SC) injection. Thus, oral administration of MEDI7219 may provide a better option for patients by increasing compliance and ease-of-use. MEDI7219 tablets formulated for oral delivery require an enteric coating to protect the peptide from degradation in the stomach, thereby ensuring that the peptide and absorption enhancers reach the small intestine in proximity to each other for optimal absorption. Oral administration of hydrophilic macromolecules has been challenging primarily due to low intestinal epithelial permeability. Challenges usually come from passive or carrier-mediated transcellular permeation across phospholipid bilayers, as well as restricted paracellular transport via tight junctions. Propyl gallate (PG) has been used as a permeation enhancer excipient in several clinical trials.^3–5^ To increase oral bioavailability, the MEDI7219 peptide was formulated as an enteric-coated (EC) tablet with a novel combination of excipients, with PG being one of the primary permeation enhancers.^6^

PG is the propyl ester of gallic acid and is a Generally Recognized As Safe (GRAS) compound used in foods, cosmetics and hair products at low levels.^7^ PG protects oils and fats in products from oxidation and has been added to foods containing oils and fats since 1948.^8^ It is estimated that PG is used in over 150 cosmetic products at a maximum concentration of 0.1%.^9^ Free radicals of PG can inhibit the activity of enzymatic hydrolysis of adenosine triphosphate (ATP).^10^ PG may also inhibit hepatic microsomal hydrolase and demethylase activities.^11^ As a food and cosmetic antioxidant, despite the presumed low toxicity, PG has been investigated to assess safety and various toxicological properties.^12–14^ The European Food Safety Authority has accepted a daily intake of 0.5 mg/kg body weight for this compound, primarily because of a gap in available toxicity data more so than because of a specific safety concern. For a 60 kg individual, the proposed oral GLP-1 formulation would contain approximately 1.3-fold more PG than stipulated in these recommendations. Furthermore, the amount of PG planned for the MEDI7219 formulation is approximately 25-fold higher than in currently approved products in the United States [CDERs inactive ingredient database: https://www.accessdata.fda.gov/scripts/cder/iig/index.cfm]. Early investigation of the toxicity profile of PG can be traced back to the 1980s and 1990s with focus on hepatic injury.^11–13^ These reports indicated that PG impairs mitochondrial function thus inducing cytotoxicity of hepatocytes. Renal injury and potential species difference from PG exposure have not been reported. In advance of the first-in-human clinical study of MEDI7219 (NCT03362593), the toxicity profile of EC MEDI7219 tablets containing the intended clinical excipients, including PG, was evaluated in dogs to supplement the literature-based safety information. Good laboratory practice (GLP) investigational new drug (IND)-enabling toxicology study 001 using the complete formulated, EC tablets in dogs showed signs of renal toxicity. As this finding was observed in the presence or absence of the GLP-1 peptide, it was suspected that one or more of the excipients included in the formulation was responsible for the toxicity. To elucidate this further, a series of tablets that included different combinations of the various excipients were tested in dogs in an initial 4-week investigational study 002. This was later followed by a second study investigating the requirement of an EC tablet to induce PG-mediated nephrotoxicity (study 003). Only EC tablets containing PG induced nephrotoxicity and a non-EC capsule containing PG alone did not induce nephrotoxicity. The combined results of these studies indicated that PG, when formulated in EC tablets, was likely inducing renal toxicity, either directly or indirectly.

Since PG has been rarely studied as an excipient for oral delivery of biotherapeutics, detailed investigations of PG pharmacokinetics, metabolism, and safety considerations have been very limited to date. To better dissect the mechanism of PG-induced nephrotoxicity we conducted in vitro studies in both human and dog renal proximal tubule epithelial cells (RPTECs). RPTECs have been used to evaluate the potential for renal toxicity, discovery of new biomarkers, and elucidation of mechanisms of nephrotoxicity in vitro.^15–17^ In order to better characterize pharmacokinetics and metabolism of PG, we developed a high sensitivity multiplexed LC-MS/MS method to quantitatively measure PG and its nine phase-I and phase-II metabolites in dog and human plasma samples, providing further understanding of PG metabolism in both species. Analytical methods such as gas and liquid chromatography have traditionally been the primary methods used to determine concentrations of PG as an antioxidant additive in foods.^18–20^ The use of LC-MS/MS for measurement of PG has not been commonly employed and has been limited to either in food application or utilizing PG as an internal standard of a pharmacologically active constituent in rat plasma.^21,22^

## Materials and Methods

### Reagents and Materials

EC tablets containing various excipients were manufactured by MedImmune (Gaithersburg, MD) as described previously.^1^ Gallic acid (GA) was purchased from Sigma (St. Louis, MO). PG was purchased from USP (Rockville, MD). 4-O-methyl gallic acid (4OMGA) was purchased from VWR Scientific (Allison Park, PA) and Extrasynthese (Lyon, France). Propyl gallate-3-glucoronide (PG-3-Gluc), propyl gallate-4-glucoronide (PG-4-Gluc), gallic acid-3-glucoronide (GA-3-Gluc), gallic acid-4-glucoronide (GA-4-Gluc), propyl gallate-glutathione (PG-GSH), gallic acid-glutathione (GA-GSH), and 4-O-methyl gallic acid-3-glucoronide (4OMGA-3-Gluc) were purchased from WuXi AppTec (Shanghai, China). Gallic acid-^13^C_5_ and 4-O-methyl gallic acid-^13^C, d_3_ were purchased from Key Organics (Camelford, UK). Propyl gallate-d_5_ was purchased from Chemtos (Austin, TX). CellTiter-Glo luminescent cell viability assay kit and GSH/GSSG-Glo assay were from Promega (Madison, WI). Human and dog RPTEC and media were from BioIVT (Westbury, NY). dog RPTECs media were purchased from Innoprot (Biscay, Spain).

Acetonitrile (HPLC grade), methanol (HPLC grade), and formic acid (LC-MS grade) were purchased from Sigma-Aldrich (St. Louis, MO). 100% filtered water was purchased from EMD Millipore (Burlington, MA). Dimethyl sulfoxide (ACS grade, ≥ 99.9%) was purchased from Honeywell (Charlotte, NC). Ammonium bicarbonate, Baker analyzed reagent (21.30–21.73%) was purchased from J.T. Baker (Phillipsburg, NJ). Dipotassium EDTA (K_2_EDTA) human plasma and dog plasma were purchased from commercial sources, such as Bioreclamation (Westbury, NY) and Biological Specialty Corporation (Colmar, PA).

### Cell Culture

Frozen RPTECs vials were removed from liquid nitrogen storage and placed in a 37 °C water bath until the contents were completely thawed. The cells were gently resuspended and dispensed into a cell culture flask at a seeding density of ∼7500 cells/cm^2^. The flask was placed in a 37 °C, 5% CO_2_ incubator. Cell cultures were given fresh medium the following morning and then every other day until the cell culture was approximately 90% confluent. When the cell culture was 90% confluent, medium was removed and the cells were rinsed with warmed PBS. Cells were harvested and split into new flasks based on a cell density of 7500 cells/cm^2^. Cells were incubated at 37 °C for 3 days, with fresh medium added each day. Cells were harvested and diluted with growth medium to a density of 200,000 cells/mL. 100 μL of cell suspension per well (20,000 cells/well) was added to a clear-bottom 96-well microplate for luminescence assays. The plated cells were incubated in the 37 °C incubator for 3 days.

### Compound Treatment of RPTEC

Each test compound (PG, GA, 4OMPG and 4OMGA) was initially reconstituted using DMSO at a concentration of 250 mg/mL. Serial dilutions of each compound were performed to reach concentrations of 4000 μM, 2000 μM, 1000 μM, 500 μM, 250 μM, 125 μM, 62.5 μM, 31.3 μM using warmed fresh supplemented medium with 0.4% (v/v) DMSO for all concentration levels. 100 μL of media containing the compound solution was added to the cells. Final concentrations of the compounds were 2000 μM, 1000 μM, 500 μM, 250 μM, 125 μM, 62.5 μM, 31.3 μM, 15.7 μM. AZ13599185 (tubulysin, 100 or 500 ng/mL) or cisplatin (30 μg/mL) and buthionine sulfoximine (BSO, 200 μM) were used as positive controls for CTG and GSH assays, respectively. 0.2% (v/v) DMSO in medium was used as the control for both assays, corrsponding to the final cocnetration of DMSO in the well. Three replicates for each compound at each concentration were prepared. Six replicates of positive or negative controls were prepared. Cells were incubated at 37 °C for approximately 48 h.

### Cell Titer Glo and Glutathione Assays

Cell media were removed and cells were washed twice with 200 μL of PBS buffer per well. CTG and GSH assay reagents were added to the wells and the assays were performed following the protocols of the Promega assay kits. Briefly, to perform the CTG assay, 100 μL of CTG solution was added into each well. The plate was gently shaken and incubated in the dark for 30 min and luminescence was measured using a PerkinElmer VICTOR X3 plate reader. To perform the GSH assay, luciferin-NT substrate and glutathione S-transferase were mixed into the GSH-GLo solution. 100 μL of the mixed solution was added into each well. The plate was gently shaken and incubated in the dark for at least 30 min. 100 μL of Luciferin Detection Reagent was added per well. The plate was gently shaken and incubated in the dark for at least 15 min. Luminescence was measured using a PerkinElmer VICTOR X3 plate reader.

### Preparation of Plasma Samples

Dog blood was collected via jugular vein in potassium (K_2_) EDTA tubes and processed to plasma. Samples were centrifuged at approximately 3000 rpm for 5 min at approximately 4 °C within 30 min of collection. Plasma samples were acidified with 1% (v/v) formic acid (FA) to avoid degradation of PG and metabolites in plasma within 20 min of the start of centrifugation and then stored frozen (≤-60 °C). Acidified plasma was extracted using internal standard-spiked solution (100 mM ammonium bicarbonate: acetonitrile = 1:4). Samples were then centrifuged to collect supernatant, followed by drying to completion. The dried samples were reconstituted with 200 uL of 0.1% (v/v) formic acid in water. Samples were vortexed and centrifuged before injection. In order to assess potential impact of hemolysis on qualification of PG and its metabolites, hemolyzed plasma was prepared by spiking fully hemolyzed whole blood at an indicated percentage into plasma.

### LC-MS/MS Analysis

Separations were conducted on a reversed-phase column (Phenomenex, Kinetex F5, 2.6 μm 100 Å, 100 × 4.6 mm) at a flow rate of 0.7 mL/min at 40 °C. Mobile phase A was 0.1% formic acid in water and mobile phase B was 0.1% formic acid in methanol. The samples were analyzed on a SCIEX 6500+ QTRAP under negative ESI mode. The total run time was 5.5 min. Eight calibration standards and three quality control (LQC, MQC and HQC) samples were used for quantification. Further details of the LC-MS/MS method are described in Supporting Information and Methods.

### Data Analysis

The targeted quantitation of PG and phase-I/II metabolites was conducted using peak area ratio of analytes and internal standards in MultiQuant software (SCIEX).

For cell-based assays, data was normalized to the negative control. Cell viability and GSH level were expressed as

$$\mathrm{Cell viability or GSH level}=\frac{\mathrm{Luminescence}\;\left(\mathrm{each well}\right)}{\mathrm{Luminescence}\;\left(\mathrm{average of DMSO negative control}\right)}$$

#### Animal Studies

The in vivo dog studies were conducted at Covance Laboratories (later renamed LabCorp Early Development Laboratories, Inc.; Madison, WI and Somerset, NJ) in compliance with the Animal Welfare Act, the Guide for the Care and Use of Laboratory Animals, and the Office of Laboratory Animal Welfare. Female beagles 6–8 months of age and weighing 6.1–9.8 kg at initiation of dosing were used. Beagles were housed in stainless steel cages on Tenderfoot flooring and were socially housed, unless individually housed during acclimation, for study related procedures, for behavior or health reasons, due to the number of animals available, or for individual assessment of food consumption. Starting on Day 2 of the dosing phase, animals were separated prior to dosing and were commingled approximately 0.5 h post the last animal dosed. Certified Canine Diet #5007 (PMI Nutrition International Certified LabDiet) was provided for 4 to 5 h each day. Feed was offered approximately 3 h following completion of dosing on days of dosing or at approximately the same time as the expected end of dosing (±2 h) on days without dosing. Water was provided ad libitum.

In the first investigative 4-week study (study 002), dogs received EC tablets consisting of different combinations of the clinical formulation excipients (Table 1) with or without PG once daily for 27 days. For groups receiving tablets with PG, each EC tablet contained 100 mg PG; the number of tablets administered each day (13–20 placed in size 12 gelatin capsules) was adjusted based on the most recent body weight to yield a target PG dose level of 200 mg/kg/day. Scheduled necropsies of all surviving animals were conducted on day 28 of the dosing phase. Samples for PG toxicokinetics were collected on days 1, 14, 21, and 25 of the dosing-phase. On days 1, 14, and 21 samples were collected: predose and approximately 1, 2, 3, 4, 6, 8, 12, and 24 h postdose. Days 25: predose and approximately 1, 2, 3, 4, 6, 8, 10, and 24 h postdose. Urine for the acute renal injury biomarker, neutrophil gelatinase-associated lipocalin (NGAL), was collected at least twice during the predose phase, on Days 8, 15, and 22 of the dosing phase, and on the day of scheduled sacrifice. Urine was collected via cystocentesis at necropsy from animals sacrificed at an unscheduled interval. NGAL was measured using an ELISA method and normalized to urine creatinine. Blood samples for standard clinical chemistry parameters, including creatinine and urea nitrogen, were collected from fasted dogs twice prior to the predose phase and before daily dose administration on Days 7, 14, and 21 of the dosing phase, as well as on the day of scheduled necropsy; samples were measured on a clinical chemistry analyzer using a validated method.

**Table 1:**
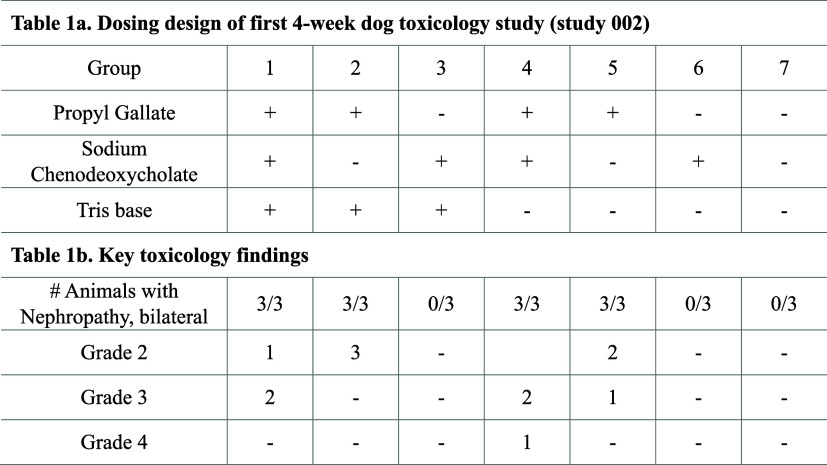
Four-Week Investigative Repeat-Dose Toxicology Study 002 with EC Excipient Tablets in Beagles^a^

a n = 3. Target dose levels of PG, NaCDC, and Tris (tris­(hydroxymethyl)­aminomethane) were 200, 100, and 38 mg/kg/day, respectively. a. Each group received one of 7 different tablet formulations with various excipient combinations of PG, NaCDC, and Tris. Other tablet components were the same for all formulations and comprised compendial excipients commonly used in clinical oral tablet formulations (mannitol, crospovidone Aerosil 300 Pharma, sodium stearyl fumarate, Eudragit PlasACRYL HTP20). b. Nephropathy was only found in animals dosed with EC tablets containing PG.

In the follow-up 4-week study (study 003), dogs received EC tablets consisting of the clinical formulation excipients minus PG, clinical formulation excipients minus NaCDC, or PG alone in a gelatin capsule once daily for 26 days. Each tablet or capsule contained 100 mg PG; the number of tablets or capsules administered each day (12–15 placed in size 12 gelatin capsules) was adjusted based on the most recent body weight to yield a target PG dose level of 200 mg/kg/day. Scheduled necropsies of all surviving animals were conducted on day 27 of the dosing phase. Samples for PG toxicokinetics were collected on days 1 and 22 of the dosing-phase at predose (Day 22 only) and 0.5, 1, 2, 4, 6, 8, 12, and 24 h postdose. Urine for NGAL measurement was collected at least twice during the predose phase, on Days 8, 15, and 22 of the dosing phase, and on the day of scheduled necropsy. Plasma samples for the measurement of lipopolysaccharide (LPS) were taken twice in the pretest period, predose and 2 h after dosing on Days 15 and 22, and prior to necropsy. LPS concentrations were measured using a qualified sandwich ELISA method (LSBiosciences) with a detection range of 7.82 to 500 pg/mL.

## Results and Discussion

### Toxicology Studies of Propyl Gallate in MEDI7219

The toxicity profile of different excipient combinations was evaluated upon daily administration of EC tablets to female beagle dogs for 27 days in study 002. Dogs were assigned across seven groups (3/group) and tablets containing different excipient combinations were administered by oral gavage in gelatin capsules once daily to determine which excipient(s) was responsible for causing the renal toxicity that had been observed in repeat-dose toxicology studies conducted with MEDI7219 EC tablets (Table 1a). Results of this investigative 4-week study of orally administered EC excipient tablets in beagles revealed PG as the possible cause of renal toxicity (Table 1b). Following oral administration of EC tablets comprising different combinations of excipients, nephrotoxicity was only observed in dogs dosed with formulations comprising PG. In a follow-up study (study 003), 200 mg/kg/day PG alone administered in standard gelatin capsules did not result in nephrotoxicity while kidney injury was observed in the group receiving EC tablets consisting of the clinical excipients minus sodium chenodeoxycholate (NaCDC; data not shown). Although in this follow-up study, PG exposure (AUC_0–24_) from the PG capsules was lower than from the EC tablet, the exposure achieved from the PG capsules was in the range at which renal toxicity was seen after ≥4-weeks of dosing with EC tablets across all dog studies conducted for the MEDI7219 toxicology program (Supplemental Table 2).

Microscopic evaluation of kidneys from animals administered PG via EC tablets showed renal nephropathy consisting of tubular degeneration and regeneration and occasionally focal interstitial infiltrates of mononuclear cells or brown-yellow pigment in tubular epithelial cells. In addition, dilation of glomeruli was observed in some affected tubules. Additionally, these animals also had an increase in urine NGAL levels, one of the 6 acute renal injury biomarkers recommended by the FDA for clinical monitoring. Time-dependent increases in NGAL levels were observed in dogs receiving EC excipient tablets with PG (Figure 1). Other excipients such as NaCDC did not induce elevations in NGAL levels. This biomarker correlated well with the histopathological nephropathy observed in these animals, as elevated NGAL levels were not observed in animals that did not have microscopic kidney injury. While some animals with nephropathy also had elevated serum creatinine and/or urea nitrogen, the correlation to microscopic injury was less consistent than urine NGAL.

**Figure 1:**
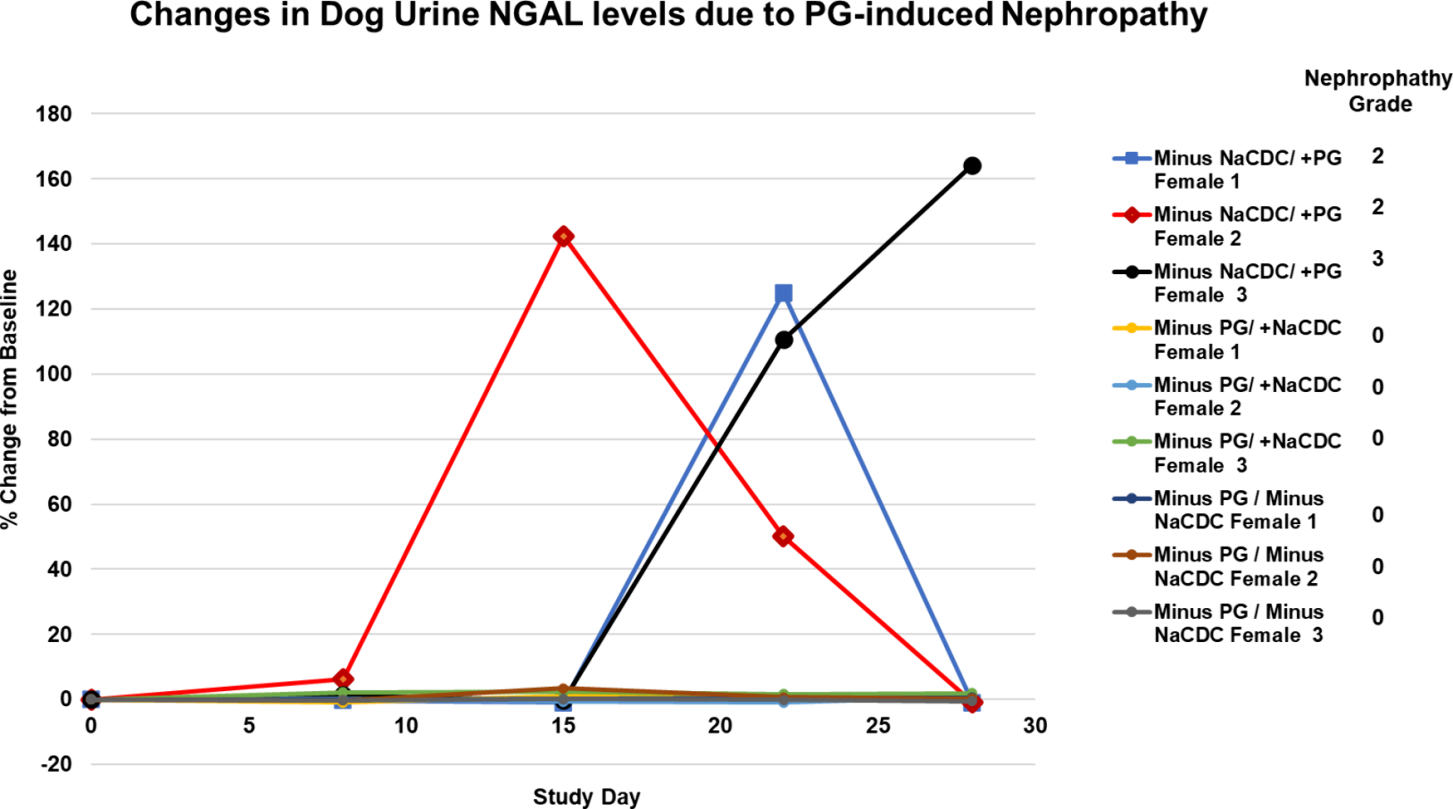
Changes in dog urine NGAL levels due to PG-induced nephropathy in study 002 (animals from Groups 5, 6, and 7). Acute renal injury biomarker - urinary NGAL increased in all animals receiving PG but not in other groups. *N* = 3 dogs per group. Individual animal profiles are shown.

Although chenodeoxycholate and other bile acids have been shown to increase intestinal permeability^26–29^ ), our colleagues demonstrated that lower oral bioavailability of MEDI7219 occurred in dogs when the ratio of NaCDC to PG was larger.^30^ The bioavailability of MEDI7219 in an EC tablet that excluded PG has not been reported, so the specific contribution of NaCDC in the absence of PG to MEDI7219 permeability is uncertain. The finding that higher amounts of PG are required for greater peptide bioavailability suggests that, in the context of this EC tablet formulation, an optimal ratio of PG:NaCDC for MEDI7219 bioavailability and perhaps overall gut permeability exists. This could explain the lack of similar renal toxicity with tablet formulations containing NaCDC without PG. Additionally, dogs administered tablets without PG were not exposed to reactive PG metabolites that may have contributed to the observed renal toxicity.

### In Vitro Assessment of PG Toxicity in Primary RPTEC

The metabolism pathway for PG has been described in detail previously.^12,23^ The proposed mechanism of toxicity for PG and GA arises from the formation of a reactive phenoxy radical species leading to the formation of an unstable o-quinone. The o-quinone then undergoes detoxification by GSH conjugation. While both PG and GA can lead to the formation of phenoxy radical and quinone intermediates, PG is much more lipophilic than GA and is expected to cross cell membranes more readily (Log P for PG is 2.13 and for GA is 0.91).^23^

Because of the potential implication of the apparent PG-mediated nephrotoxicity on the clinical safety of MEDI7219, the mechanism of dog renal toxicity and its translatability to humans was evaluated in human and dog RPTECs via in vitro assessments of PG and its metabolites GA and 4OMGA. A concentration-dependent decrease in cell viability was observed with PG for both dog and human RPTECs (Figure 2). The relative difference in sensitivity for PG between human (EC_50_ ∼ 1529 μM) and dog (EC_50_ ∼ 46.5 μM) was estimated to be ∼33-fold, indicating that dogs may be more sensitive to PG-induced renal toxicity than humans in vivo. PG was more cytotoxic for dog RPTEC than its metabolites, GA and 4OMGA, as well as 4-O-methyl propyl gallate (4OMPG), a synthetic analog of PG that is unable to form a reactive o-quinone. Compound structures are depicted in Supplemental Figure 1.

**Figure 2:**
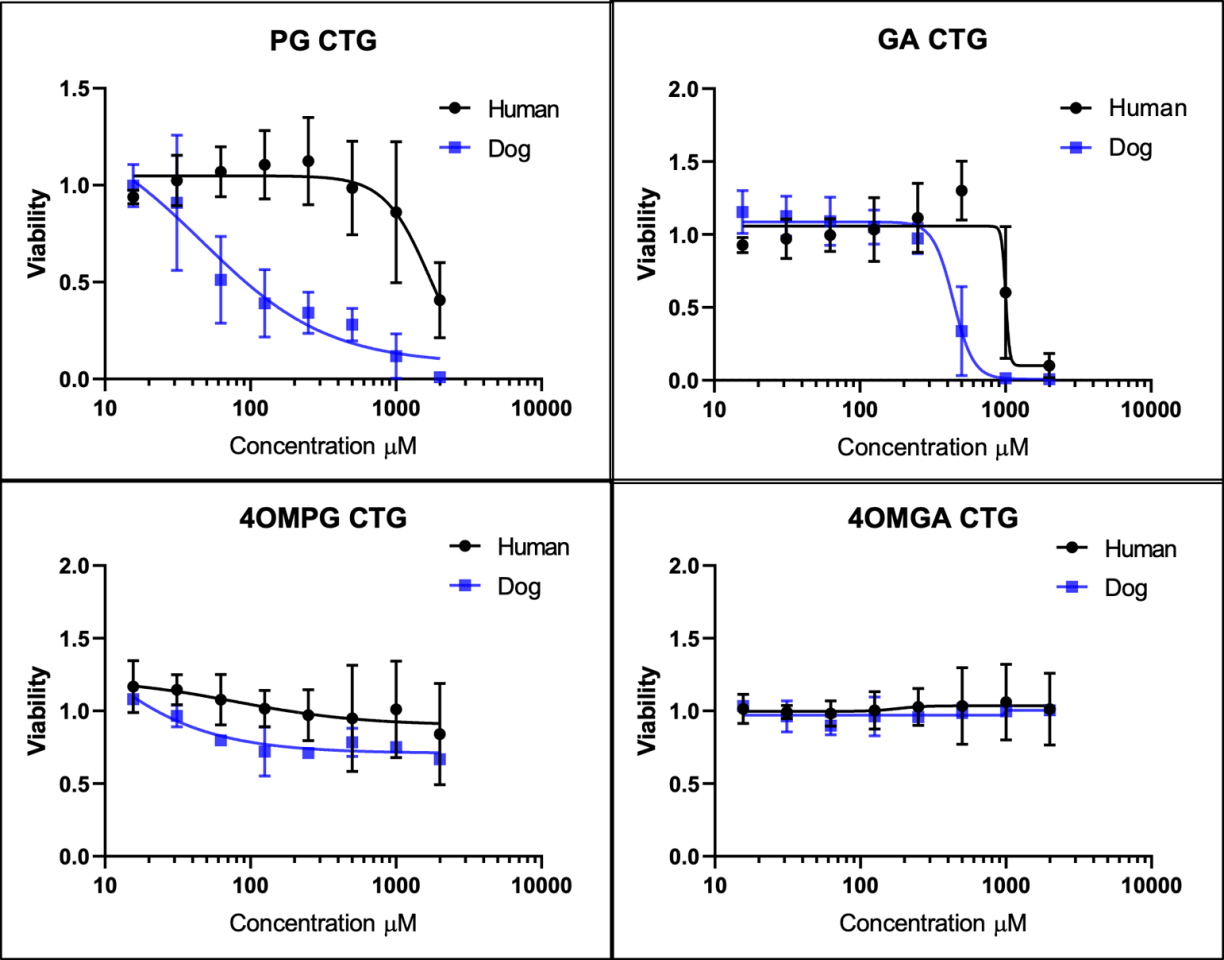
Cytotoxicity estimations in dog and human RPTECs after 48 h exposure to PG, GA, 4-O-methy-propyl gallate (4OMPG), and 4-O-methyl gallic acid (4OMGA). All human RPTEC determinations were from 5 independent experiments (2 different donors). For dog RPTECs PG and GA determinations were from 5 independent experiments, for 4OMPG and 4OMGA determinations were from 3 independent experiments (2 different donors). All experiments had 3 replicates per experiment. 4OMPG was used as a control compound.

The hypothesis that dog cells are more sensitive to PG than human cells due to PG-mediated glutathione depletion was also studied. The results show that glutathione levels were induced by PG in human but not in dog cells, suggesting that GSH-mediated detoxification of the reactive quinone of PG may be less effective in dog RPTEC than human RPTEC (Figure 3). Recent studies indicate that while dog and human glutathione-s-transferase (GST) show substantial similarity some differences in activity toward some substrates such as isothiocyanates exist with dog GST being less active than human.^24^ High concentrations of PG and GA induced a reduction of GSH levels in both dog and human cells, which was likely due to decreased cell numbers at these high concentrations of PG and GA. High concentrations of 4OMPG also led to increases of GSH in human cells, possibly due to demethylation of 4OMPG.

**Figure 3:**
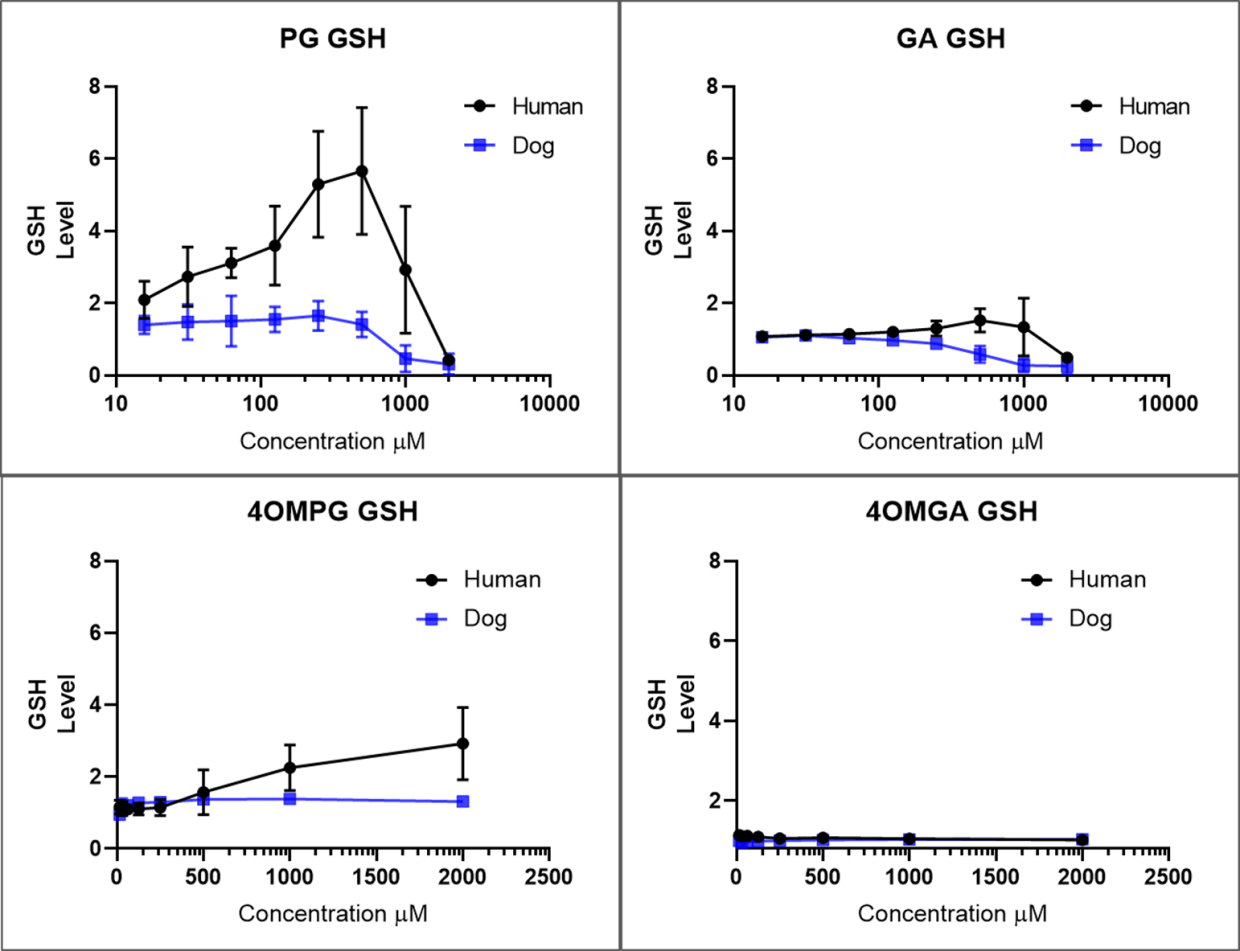
Relative GSH levels in dog and human RPTECs after ∼48 h exposure to PG, GA, 4OMPG, and 4OMGA. All human RPTEC determinations were from 5 independent experiments (2 different donors). For dog RPTECs PG and GA determinations were from 5 independent experiments (2 different donors), for 4OMPG and 4OMGA determinations were from respectively 2 and 3 independent experiments (1 donor). All experiments had 3 replicates per experiment. PG induces glutathione (GSH) levels in human cells, but not in dog cells. All data (luminescence (CPS)) was normalized to DMSO-treated negative control (luminescence (CPS)) for each donor for each experiment.

### Development of LC-MS/MS Assay for PG and Its Phase-I and Phase-II Metabolites in Dog and Human Plasma

LC-MS/MS analysis of PG and its metabolites in both dog and human plasma was performed to further understand the potential differences of PG disposition and its metabolism between humans and dogs. A highly sensitive 10-plex method for the quantification of PG and its phase-I and phase-II metabolites was developed. Figure 4 shows a chromatogram of all targeted metabolites and an example of PG assay performances including PG intra/inter assay quality controls in human plasma, demonstrating appropriate precision and accuracy. The 3-plexed method of PG and its phase-I metabolites (GA and 4OMGA) was fully validated per 2018 FDA guidance,^18^ with LLOQ of propyl gallate at 20 pg/mL in human plasma and 40 pg/mL in dog plasma. Other major metabolites such as glutathione and glucuronide conjugates were detected at low nanogram levels, which were sufficient for quantifying the study samples. Table 2 summarizes LLOQ of each analyte.

**Figure 4:**
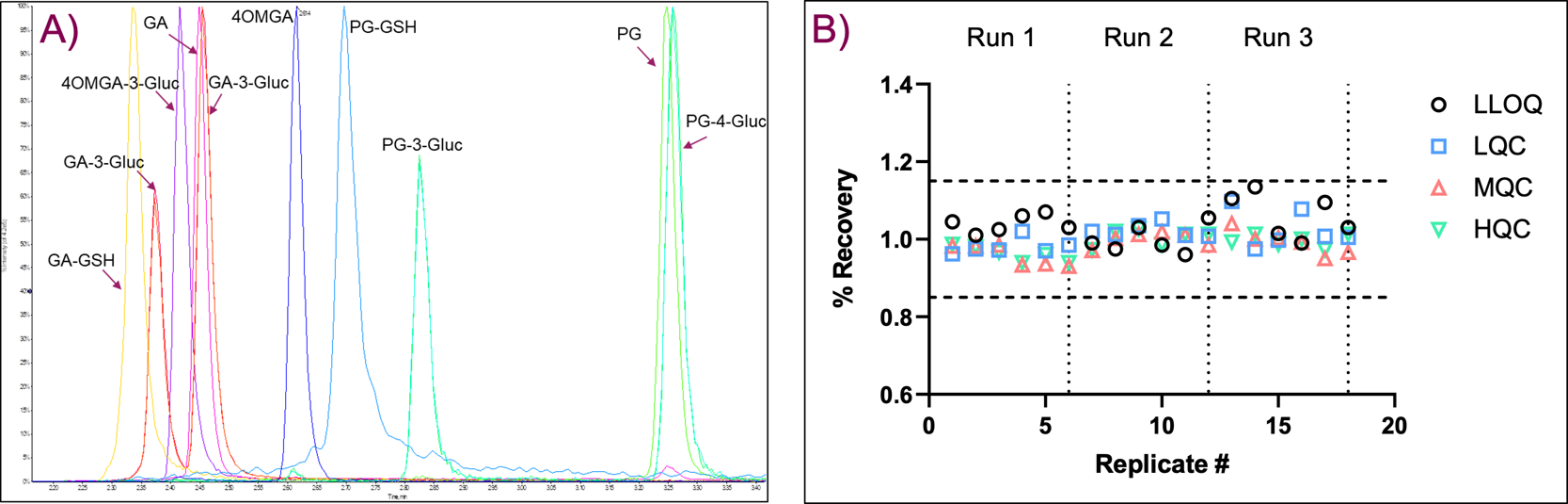
A). A representative chromatogram of PG and its nine phase-I and phase-II metabolites standards in 0.1% formic acid in water. B) PG intra/inter assay quality controls in human plasma demonstrating acceptable assay precision and accuracy. GA and 4OMGA had similar performances.

**Table 2:** Summary of LLOQ of Each Analyte^a^

Compound	LLOQ in human plasma (ng/mL)	LLOQ in dog plasma (ng/mL)
Propyl gallate (PG)*	0.02	0.04
Gallic acid (GA)*	1	2
4-O-methyl gallic acid (4OMGA)*	5	3
Propyl gallate-glutathione (PG-GSH)	1	
Gallic acid-glutathione (GA-GSH)	10	
Propyl gallate-3-glucoronide (PG-3-Gluc)	0.2	
Propyl gallate-4-glucoronide (PG-4-Gluc)	0.2	
Gallic acid-3-glucoronide (GA-3-Gluc)	10	
Gallic acid-4-glucoronide (GA-4-Gluc)	10	
4-O-methyl gallic acid-3-glucoronide (4OMGA-3-Gluc)	8	

a An * indicates assays of these analytes were validated in both human and dog plasma.

Unforeseen matrix interferences, that were not observed in predose or placebo samples, were observed in plasma samples upon administration of PG. Therefore, the quantitative measurement of PG and 4OMGA from pharmacokinetic samples was confounded by matrix interferences. To address the method limitations due to interference, extensive chromatographic optimization involved evaluation of columns, gradients, mobile phases, flow rate and column temperature to resolve the analyte peak from matrix interferences (Supplemental Figure 2). An impact of hemolysis on PG and GA quantification was also observed even at such low levels as 0.05%. However, their respective internal standards also showed similar response as the analytes. As a result, the impact of hemolysis on PG quantification can be controlled with a stable labeled internal standard (Supplemental Figure 3).

### Pharmacokinetic Assessment of Propyl Gallate in Humans Using Validated Method

Healthy volunteers participating in the clinical study NCT03362593 received single or multiple ascending doses of MEDI7219 tablets. Healthy volunteers received EC MEDI7219 tablets containing 200, 400, or 800 mg PG per tablet. The clinical plasma samples were analyzed using the validated method described above. Figure S4 shows representative PG pharmacokinetic data. After oral administration of tablets containing PG, the *T*_max_ of PG was reached around 3–4 h postdose. PG exposure (mean AUC_0‑t_ and *C*_max_) generally increased over the study duration with increasing dose levels.

### Pharmacokinetic Assessment of Propyl Gallate and Its Metabolites in Human and Dog

The primary metabolites of PG were measured in dog and human plasma following oral administration of EC MEDI7219 tablets to examine a potential mechanism of the PG-mediated renal toxicity in dog and its possible translatability to humans. Plasma concentrations of PG, its phase-I metabolites GA and 4OMGA and phase-II glucuronide and glutathione conjugates were measured. The human and dog samples, collected through 8 h after administration, were analyzed and the results were compared (Figure 5, Figure S5). Based on allometric scaling method the highest daily dose administered in humans was approximately 8-fold less compared with the dose level tested in dogs. However, at these doses the observed exposures to PG, GA or 4OMGA were comparable between the two species, thus suggesting that humans may have greater oral bioavailability of PG than dogs. However, to our knowledge, PG exposure data following IV administration is not available for either humans or dogs and therefore a potential species difference in PG bioavailability cannot be confirmed. The metabolism pattern between the two species was largely similar with some differences observed in exposure of PG glucuronides and GA glucuronides. PG glucuronides had higher exposure while GA-3-glucoronides showed lower exposure in humans than in dogs. Both PG-GSH and GA-GSH conjugates had very low exposure in circulation with potential minor differences between humans and dog observed.

**Figure 5:**
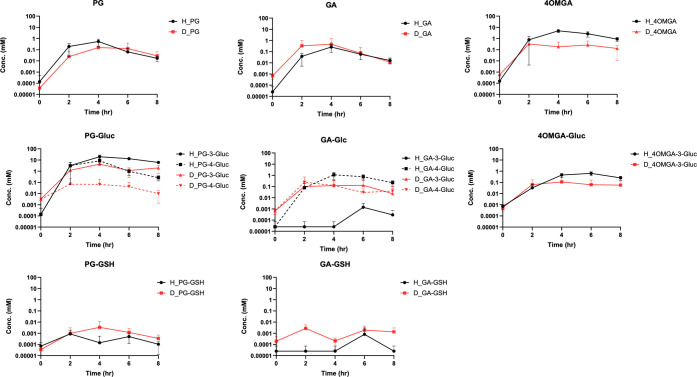
Exposure comparison of PG and phase-I and phase-II metabolites in dog and human plasma. Human (H) data is in black. Dog (D) data is in red (study 002). Concentration (mM) is in log10 scale. Data was normalized to PG dose (mg/kg/day). *N* = 8 for human (PG dosed at 800 mg with MEDI7219 tablets). *N* = 6 for dogs (PG dosed at 200 mg/kg/day). Below LLOQ values were set to 1/2 LLOQ for the calculation. (Compound abbreviations in figures are summarized in Table 2).

## Conclusion

Oral delivery, as a noninvasive drug administration route for peptides and biologics, has been of growing interest for the development of chronically administered medications since it can significantly improve the patient experience. Novel design of formulation is one of the means to enhance drug properties such as permeability and solubility. Propyl gallate, an excipient commonly used in food and cosmetics to avoid oxidation, has been recently found to enhance the bioavailability of a GLP-1 agonist, MEDI7219, when formulated as an EC tablet.^30^ As a novel excipient (i.e., used at higher levels than in approved oral medications), pharmacokinetic and toxicokinetic measurements of PG and its metabolites are important to establish exposure-based safety margins. Preclinical toxicology studies in dogs have revealed that PG delivered via EC tablets induced renal toxicity. In vitro assessments using RPTEC identified a possible sensitivity difference between dogs and humans, providing further understanding of potential translatability of PG-induced dog renal toxicity to humans. Mechanistic investigations by cell-based assays with RPTEC suggested GSH-mediated detoxification was less effective in dog cells than in human cells. A highly multiplexed, robust and sensitive LC-MS/MS-based method was developed for the quantification of PG and its nine phase-I and phase-II metabolites in both dog and human plasma. The methods were evaluated and validated comprehensively to support evaluation of PG safety and pharmacokinetics. Species differences in PG drug metabolism at a molecular level were further investigated using the LC-MS/MS assay. Some exposure differences in phase-II metabolites of PG were observed in circulation between humans and dogs. We also observed a difference in overall PG exposure, assuming allometric scaling.

Because nephrotoxicity did not occur when PG was administered in an immediate release capsule, it seems unlikely that PG is solely responsible for the renal injury observed in dogs when receiving PG via EC tablets. One hypothesis is that while PG enhances the gastrointestinal permeability of the MEDI7219 peptide, it may also allow other substances to cross the gastrointestinal membrane, such as bacterial-derived inflammatory factors and/or uremic toxins. In the follow-up dog study, LPS was not detected in plasma (data not shown); however, it is possible that the method was not sensitive enough to detect physiologically relevant levels of LPS or the time points for plasma collection in relation to tablet dosing were not ideal for LPS detection in the context of assessing the potential impact of LPS on nephrotoxicity. Impaired gut barrier function has been shown to be associated with acute kidney injury^32^ and leaky gut has been implicated as a source of inflammation in chronic kidney disease in humans.^25^ It is possible that enhanced gut permeability by PG in conjunction with a higher sensitivity to compounds requiring GSH-mediated detoxification may be responsible for the nephrotoxicity observed in dogs receiving PG via EC tablets. However, the overall relevance of this finding for humans taking EC tablets that contain PG or other absorption enhancers is currently uncertain. Further studies are required to better understand the exact mechanism of PG-induced nephrotoxicity in dogs, the contribution of EC to intestinal membrane disruption, as well as potential species differences. Additionally, nontargeted proteomics, lipidomic and metabolomics approaches can be employed to gain more comprehensive understanding of EC PG-induced dog renal toxicity.

## Supplementary Material



## Notes

Detailed methods; chromatographic resolution of unforeseen interferences; hemolysis impact; PG pharmacokinetics in humans; linear scale comparison of PG and metabolites pharmacokinetics between dogs and humans; PG exposure comparison across dog studies (PDF)

## ¶

Anton I. Rosenbaum, Vera Therapeutics, 2000 Sierra Point Parkway, Brisbane, CA 94005, USA; anton.rosenbaum.phd@gmail.com; anton.rosenbaum@veratx.com

## References

[1] Pechenov S., Revell J., Will S., Naylor J., Tyagi P., Patel C., Liang L., Tseng L., Huang Y., Rosenbaum A. I., Balic K., Konkar A., Grimsby J., Subramony J. A. (2021). Development of an orally delivered GLP-1 receptor agonist
through peptide engineering and drug delivery to treat chronic disease. Sci. Rep.

[2] Tyagi P., Patel C., Gibson K., MacDougall F., Pechenov S. Y., Will S., Revell J., Huang Y., Rosenbaum A. I., Balic K., Maharoof U., Grimsby J., Subramony J. A. (2023). Systems Biology and Peptide Engineering to Overcome
Absorption Barriers for Oral Peptide Delivery: Dosage Form Optimization
Case Study Preceding Clinical Translation. Pharmaceutics.

[3] A Study of LY2484595 in Healthy Subjects. ClinicalTrials.gov identifier: NCT01450098.

[4] Evaluation of 2 Oral Doses of PG-760564 in Rheumatoid Arthritis (RA) Patients Receiving Methotrexate. ClinicalTrials.gov identifier: NCT00369928.

[5] Study of CGC-11047 (PG-11047) in Subjects With Advanced Refractory Solid Tumors. ClinicalTrials.gov identifier: NCT00705653.

[6] A 6-Part Study In Healthy Volunteers To Evaluate Safety, Tolerability and Uptake Of MEDI7219 in the Body When Given as Single and Multiple Doses. ClinicalTrials.gov identifier: NCT03362593.

[7] GRAS Substances (SCOGS) Database. https://www.fda.gov/food/generally-recognized-safe-gras/gras-substances-scogs-database (accessed 20 August 2024).

[8] Final report on the amended safety assessment of Propyl Gallate. Int. J. Toxicol. 2007, 26 Suppl 3, 89–118. 10.1080/10915810701663176. 18080874

[9] Fiume M. M., Heldreth B., Bergfeld W. F., Belsito D. V., Hill R. A., Klaassen C. D., Liebler D., Marks J. G., Shank R. C., Slaga T. J., Snyder P. W., Andersen F. A. (2012). Final report
of the Cosmetic Ingredient Review Expert Panel on the safety assessment
of dicarboxylic acids, salts, and esters. Int.
J. Toxicol.

[10] Brzhevskaia O. N., Kaiushin L. P., Nedelina O. S. (1966). On the existence
of free radicals
in the enzymatic hydrolysis of adenosine triphosphate (ATP). Biofizika.

[11] Depner M., Kahl G. F., Kahl R. (1982). Influence
of gallic acid esters on
drug-metabolizing enzymes of rat liver. Food
Chem. Toxicol..

[12] Nakagawa Y., Nakajima K., Tayama S., Moldeus P. (1995). Metabolism
and cytotoxicity
of propyl gallate in isolated rat hepatocytes: effects of a thiol
reductant and an esterase inhibitor. Mol. Pharmacol..

[13] Nakagawa Y., Tayama S. (1995). Cytotoxicity of propyl
gallate and related compounds
in rat hepatocytes. Arch. Toxicol..

[14] National
Toxicology P. (1982). NTP Carcinogenesis Bioassay of Propyl Gallate (CAS
No. 121-79-9) in F344/N Rats and B6C3F1Mice (Feed Study). Natl. Toxicol Program Tech Rep. Ser..

[15] Bejoy J., Qian E. S., Woodard L. E. (2022). Tissue
Culture Models of AKI: From
Tubule Cells to Human Kidney Organoids. J. Am.
Soc. Nephrol.

[16] Mody H., Nair S., Rump A., Vaidya T. R., Garrett T. J., Lesko L., Ait-Oudhia S. (2024). Identification of Novel and Early
Biomarkers for Cisplatin-induced Nephrotoxicity and the Nephroprotective
Role of Cimetidine using a Pharmacometabolomic-based Approach Coupled
with In Vitro Toxicodynamic Modeling and Simulation. J. Pharm. Sci..

[17] Nusair S. D., Abandah B., Al-Share Q. Y., Abu-Qatouseh L., Ahmad M. I. A. (2023). Toxicity induced by orellanine from
the mushroom Cortinarius
orellanus in primary renal tubular proximal epithelial cells (RPTEC):
Novel mechanisms of action. Toxicon.

[18] Page B. D. (1993). Liquid
chromatographic method for the determination of nine phenolic antioxidants
in butter oil: collaborative study. J. AOAC
Int..

[19] Page B. D. (1983). High performance
liquid chromatographic determination of seven antioxidants in oil
and lard: collaborative study. J. Assoc. Anal.
Chem..

[20] Kline D. A., Joe F. L., Fazio T. (1978). A rapid gas-liquid
chromatographic method for the multi-determination of antioxidants
in fats, oils and dried food products. J. Assoc.
Anal. Chem..

[21] Tsuji S., Nakano M., Terada H., Tamura Y., Tonogai Y. (2005). Determination
and confirmation of five phenolic antioxidants in foods by LC/MS and
GC/MS. Shokuhin Eiseigaku Zasshi.

[22] Gao S., Zhan Q., Li J., Yang Q., Li X., Chen W., Sun L. (2010). LC-MS/MS method
for the simultaneous
determination of ethyl gallate and its major metabolite in rat plasma. Biomed Chromatogr.

[23] Galati G., Lin A., Sultan A. M., O’Brien P. J. (2006). Cellular and in vivo hepatotoxicity
caused by green tea phenolic acids and catechins. Free Radic Biol. Med..

[24] Ismail A., Lewis E., Sjodin B., Mannervik B. (2021). Characterization
of Dog Glutathione Transferase P1–1, an Enzyme Relevant to
Veterinary Medicine. Int. J. Mol. Sci..

[25] Lau W. L., Kalantar-Zadeh K., Vaziri N. D. (2015). The gut as a source of inflammation
in chronic kidney disease. Nephron.

[26] Chadwick V. S., Gaginella T. S., Carlson G. L., Debongnie J. C., Phillips S. F., Hofmann A. F. (1979). Effect
of molecular structure on
bile acid-induced alterations in absorptive function, permeability,
and morphology in the perfused rabbit colon. J. Lab. Clin. Med..

[27] Freel R. W., Hatch M., Earnest D. L., Goldner A. M. (1983). Role of tight-junctional
pathways in bile salt-induced increases in colonic permeability. Am. J. Physiol..

[28] Erickson R. A., Epsten R. M. (1988). Oral chenodeoxycholic acid increases
small intestinal permeability to lactulose in humans. Am. J. Gastroenterol..

[29] Fasano A., Budillon G., Guandalini S., Cuomo R., Parrilli G., Cangiotti A. M., Morroni M., Rubino A. (1990). Bile acids reversible
effects on small intestinal permeability. Dig.
Dis. Sci..

[30] Tyagi P., Patel C., Gibson K., MacDougall F., Pechenov S. Y., Will S., Revell J., Huang Y., Rosenbaum A. I., Balic K., Maharoof U., Grimsby J., Subramony J. A. (2023). Systems biology and peptide engineering
to overcome
absorption barriers for oral peptide delivery: Dosage form optimization
case study preceding clinical translation. Pharmaceutics.

[32] Zhang J., Ankawi G., Sun J., Digvijay K., Yin Y., Rosner M. H., Ronco C. (2018). Gut-kidney
crosstalk in septic acute
kidney injury. Crit Care.

