# Supplementary material for: Investigations of Enteric-Coated Tablet Propyl Gallate-Induced Nephrotoxicity in Beagles as well as Human and Dog Renal Proximal Tubule Epithelial Cells
Source: ACS Pharmacol Transl Sci. 2025 Apr 4;8(5):1282–91. doi: 10.1021/acsptsci.4c00563 (PMC12070228; doi:10.1021/acsptsci.4c00563)
Supplement: Supplementary file 1 [file pt4c00563_si_001.pdf]

Supporting Information

Investigations of Enteric-Coated Tablet Propyl Gallate-induced Nephrotoxicity in Beagles as well as Human and Dog Renal Proximal Tubule Epithelial Cells

Si Mou[1], B. Timothy Hummer[2], Jiaqi Yuan[1], Yue Huang[1], Meina Liang[1], Raffaella Faggioni[3], Lorin K. Roskos[3], Anton I. Rosenbaum[1*§]

[1]Integrated Bioanalysis, Clinical Pharmacology & Safety Sciences, R&D, AstraZeneca, South San Francisco, CA

[2]Cardiovascular, Renal and Metabolic Safety, Clinical Pharmacology & Safety Sciences, R&D, AstraZeneca, Gaithersburg, MD

[3]Clinical Pharmacology & Safety Sciences, R&D, AstraZeneca, South San Francisco, CA

*Corresponding Author:
Anton I. Rosenbaum - 121 Oyster Point Blvd, South San Francisco, CA 94080, USA; Tel: +1-650-379-3099; E-mail: anton.rosenbaum@astrazeneca.com

§ Present address:

Anton I. Rosenbaum, Vera Therapeutics, 2000 Sierra Point Parkway, Brisbane, CA 94005, USA; anton.rosenbaum.phd@gmail.com; anton.rosenbaum@veratx.com

Supporting Methods:

LC-MS/MS analysis

The Shimadzu (Columbia, MD) LC-30AD binary pumps, SIL-30AD autosampler, and SCIEX (Framingham, MA) 6500+ triple quadrupole mass spectrometer were used for LC-MS analysis. The separation was performed on a Phenomenex (Torrance, CA) Kinetex F5 100 x 4.6 mm, 2.6 µm column with the flow rate of 0.7 mL/min and the column temperature of 40 °C. 0.1% formic acid in water was used as mobile phase A, and 0.1% formic acid in methanol as mobile phase B. The autosampler temperature was set at 8 °C. The injection volume was 10 µL (except for 7.5 µL used in PG analysis in dog plasma). The following gradient conditions were used for separations of propyl gallate and phase II metabolites in human and dog plasma: 0.00–0.50 min, isocratic 10% B; 0.50–1.00 min, linear increase from 10% to 40% B; 1.00–3.10 min, linear increase from 40%

to 80% B; 3.10–3.30 min, isocratic 80% B; 3.30–3.90 min, linear increase from 80% to 90% B; 3.90–4.10 min, isocratic 90% B; 4.10–4.30 min, linear decrease from 90% to 10% B; 4.30–5.50 min, isocratic 10% B. Separation of gallic acid and 4-O-methyl gallic acid was similar to the previous gradient conditions, except for 0.50–1.00 min linear increase from 10% to 35% B, and 1.00–3.10 min linear increase from 35% to 80% B;

The samples were analyzed in multiple reaction monitoring (MRM) mode, and the mass spectrometric parameters can be found in **Table S1** at the following conditions: ion spray voltage -3.5 kV; source temperature, 500 °C; nebulizer gas GS1 60 and GS2 80; curtain gas 35, and collision gas 8. The instrument operation and data collection were done in Analyst (AB Sciex, Framingham, MA), and the data analysis and peak integration were done in MultiQuant with 1/concentration weighted applied to calibration curve linear regression.

**Analyte**		**Q1**	**Q3**	**DP**	**CE**
Phase I metabolites (validated)	Gallic Acid	169.0	125.0	-65	-21
	Gallic Acid-$^{13}C_5$	174.0	129.0	-65	-21
	4-O-Methyl Gallic Acid	182.9	123.9	-65	-21
	4-O-Methyl Gallic Acid-$^{13}C,d_3$	187.0	124.0	-65	-21
	Propyl Gallate	211.0	124.0	-85	-32
	Propyl Gallate-d_5	216.0	124.0	-85	-32
Phase II metabolites (non-validated)	Propyl gallate-3-glucoronide	387.0	124.0	-50	-60
	propyl gallate-4-glucoronide	387.0	124.0	-50	-60
	gallic acid-3-glucoronide	345.0	169.1	-60	-28
	gallic acid-4-glucoronide	345.0	169.0	-40	-28
	propyl gallate-glutathione	516.0	272.0	-55	-26
	gallic acid-glutathione	474.0	200.8	-45	-36
	4-O-methyl gallic acid-3-glucoronide	359.0	167.8	-38	-34

Table S1. Tandem mass spectrometry MRM transitions AB SCIEX 6500+.

Supporting Data

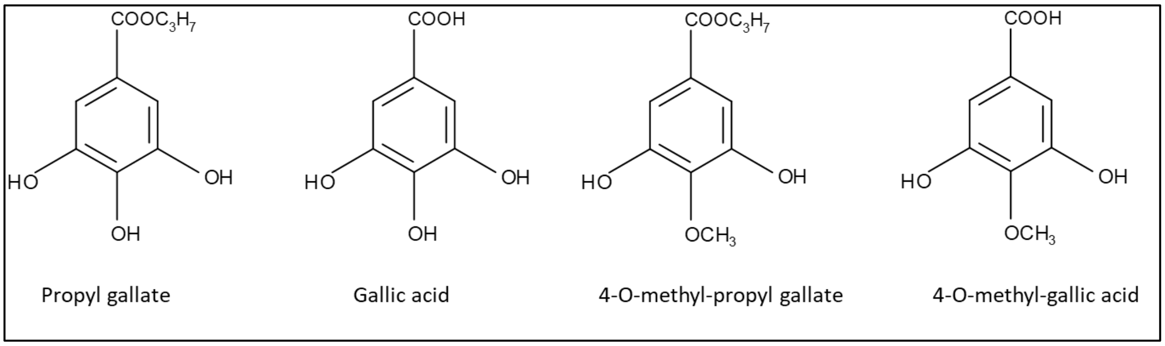

Figure S1 Structures of PG, GA, 4OMGA and 4OMPG

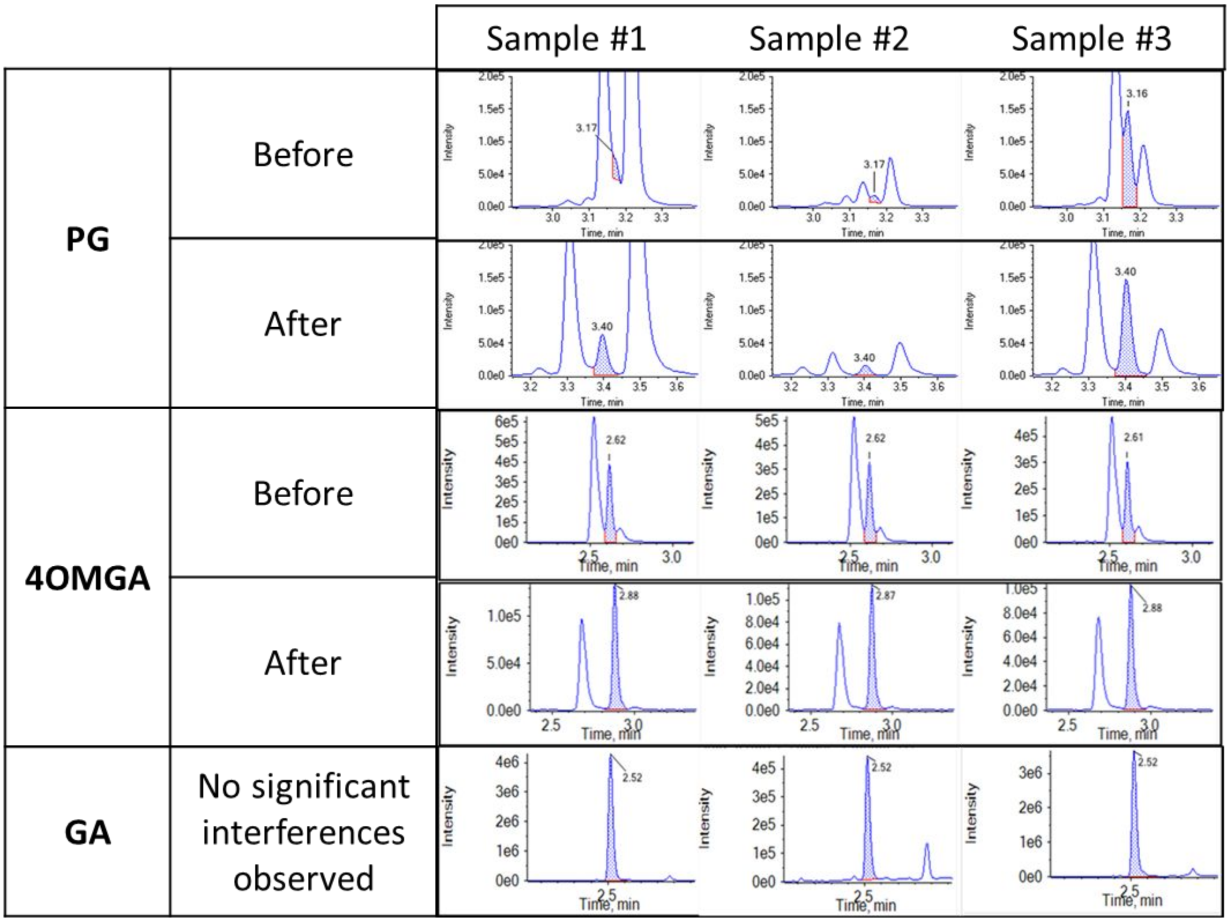

Figure S2 Chromatographic resolution of unforeseen interferences in incurred PK dog samples.

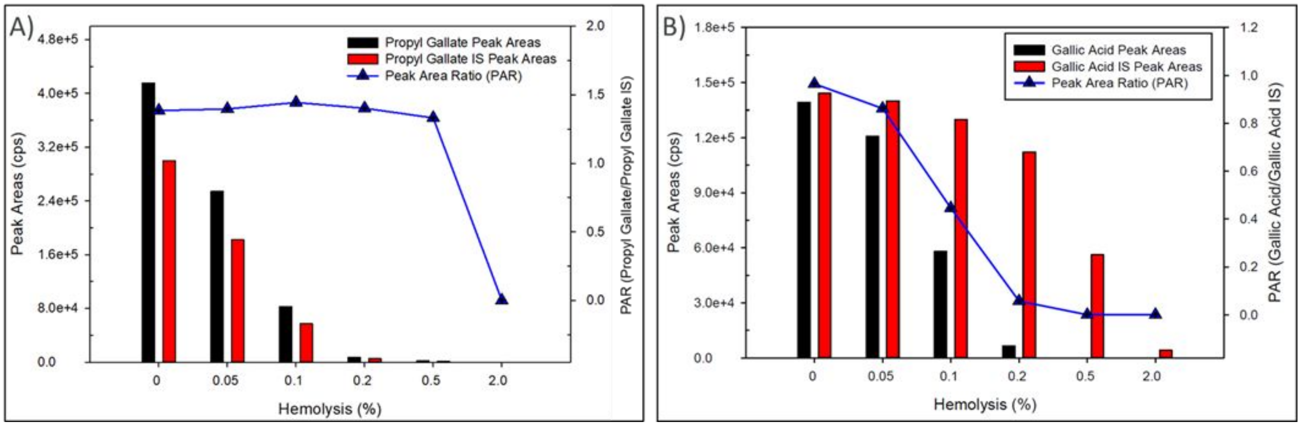

Figure S3 The response of PG/GA and its stable labelled internal standard in dog plasma samples of various degrees of hemolysis, 0 – 2%. PG and GA are sensitive to hemolysis at as low as 0.05%. However, their respective internal standards also showed similar response as the analytes. As a result, the impact of hemolysis on propyl gallate quantification can be controlled with a stable labeled internal standard.

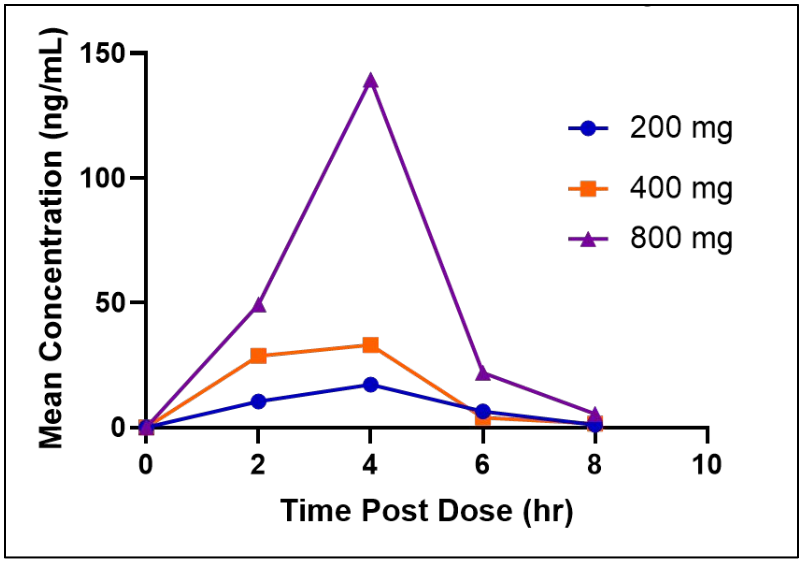

Figure S4 PG pharmacokinetic profile in a single ascending dose clinical study (NCT03362593) with oral administration of EC MEDI7219 tablets, resulting in PG doses of 200, 400, or 800 mg. LLOQ of PG is 0.02 ng/mL. The T_{max} of PG was reached around 3-4 hours post-dose.

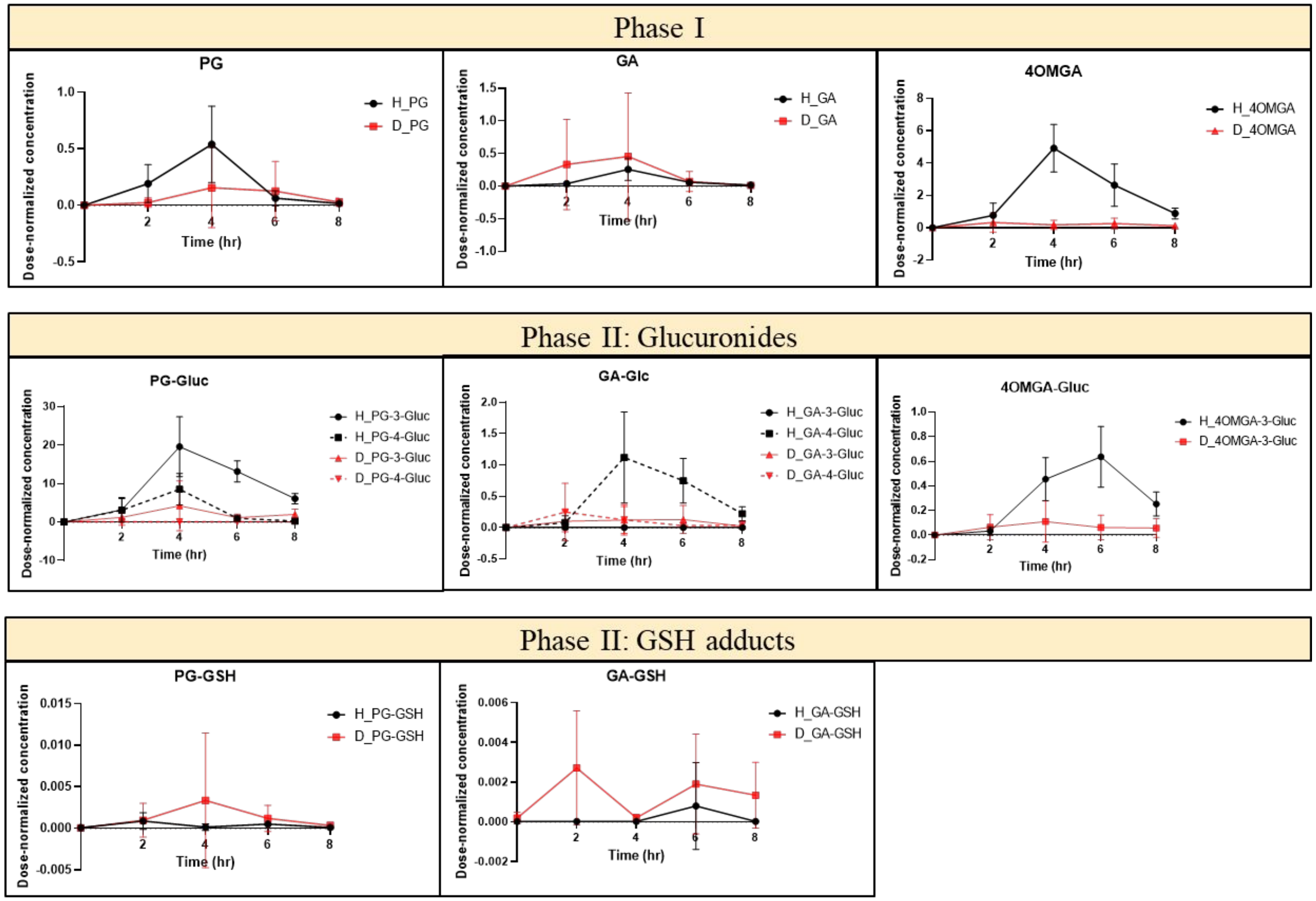

2

Figure S5 Linear scale exposure comparison of PG and phase-I and phase-II metabolites in dog and human plasma. Human (H) data is in black. Dog (D) data is in red. Data was normalized to PG dose (mg/kg/day). N=8 for human (PG dosed at 800 mg with MEDI7219 tablets). N=6 for dogs (PG dosed at 200 mg/kg/day). Below LLOQ values were set to ½ LLOQ for the calculation. (Compound abbreviations in figures are summarized in Table 2).

Daily Dose (mg/kg/day)			Formulation [a]	Number of tablets or capsules per dog per day	Propyl Gallate Exposure (AUC_{0-24}; ng·hr/mL)							
					Study 001 [b]	Study 002 [c]		Study 003 [d]	Study 004 [e]	Study 005 [f]		
PG	NaCDC	MEDI7219			Day 28	Day 14	Day 21	Day 22	Day 78	Day 112	Day 196	Day 266
12.5	10	0.75	clin excipients	2-3						72	88	90
14	7	0.4	clin excipients	1-2					101			
25	12.5	1.5	clin excipients	2-3	NR							
25	20	0.25	clin excipients	5-7						135	232	158 (M)<br>986 (F)
36	18	1.1	clin excipients	3-5					282			
44	35	2.6	clin excipients	6-9							1870	
44	22	0	clin excipients	8-11							864	
60	48	3.6	clin excipients	9-15						829		
60	48	0	clin excipients	12-14						3610		
76	38	0.75	clin excipients	6-8	618 [h]							
100	50	3	clin excipients	8-12					767			
100	50	0	clin excipients	8-11					1830			

Daily Dose (mg/kg/day)				Number of tablets or capsules per dog per day	Propyl Gallate Exposure (AUC_{0-24}; ng·hr/mL)							
					Study 001 [b]	Study 002 [c]		Study 003 [d]	Study 004 [e]	Study 005 [f]		
PG	NaCDC	MEDI7219	Formulation [a]		Day 28	Day 14	Day 21	Day 22	Day 78	Day 112	Day 196	Day 266
200	100	2	clin excipients	15-23	1210							
200	100	0	clin excipients	15-20	2560 [g]							
200	0	0	clin excipients minus NaCDC	12-15				6290				
200	100	0	clin excipients	13-19		3250	NR					
200	0	0	clin excipients minus NaCDC	14-20		2580	1850					
200	100	0	clin excipients minus Tris	14-20		2760	1800					
200	0	0	clin excipients minus NaCDC and Tris	14-18		1890	4840					
200	0	0	gelatin capsule (PG only)	13-15				1980				

Table S2. Correlation of PG exposure with nephrotoxicity.

$AUC_{0\text{-}24}$ = area under the concentration versus time curve from time 0 to 24 hours; clin = clinical; F = female; M = male; NR = no result due to insufficient data. Shaded values indicate PG exposures that resulted in microscopic nephropathy in at least 1 animal from that dose group.

[a] Clinical formulation excipients included sodium chenodeoxycholate (Na CDC), propyl gallate (PG), tris(hydroxymethyl)aminomethane (Tris)(except for Study 005), mannitol, crospovidone, aerosil, sodium stearyl fumarate, eudragit, and plasarcryl.

[b] Study 001: Initial 4-week investigational new drug (IND)-enabling good laboratory practice (GLP) study

[c] Study 002: Investigative 4-week study with different excipient combinations

[d] Study 003: Follow-up investigative 4-week study with non-enteric coated capsule

[e] Study 004: 3-month GLP study

[f] Study 005: 9-month GLP study

[g] Day 7 AUC value is presented; AUC could not be calculated for Day 28 samples due to insufficient data.

[h] Minimal nephropathy observed in 2 recovery (4 weeks) group females, but not in main group animals.
